# Bacteriophage Sf6 host range mutant that infects *Shigella flexneri* serotype 2a_2_ strains

**DOI:** 10.1093/femsle/fnac020

**Published:** 2022-02-26

**Authors:** Min Yan Teh, Elizabeth Ngoc Hoa Tran, Renato Morona

**Affiliations:** School of Biological Sciences, Department of Molecular and Biomedical Science, Research Centre for Infectious Diseases, University of Adelaide, Adelaide 5005, Australia; School of Biological Sciences, Department of Molecular and Biomedical Science, Research Centre for Infectious Diseases, University of Adelaide, Adelaide 5005, Australia; School of Biological Sciences, Department of Molecular and Biomedical Science, Research Centre for Infectious Diseases, University of Adelaide, Adelaide 5005, Australia

**Keywords:** bacteriophage, Sf6, *Shigella flexneri*, serotype 2a, host range mutant, tailspike protein

## Abstract

*Shigella flexneri* serotype 2a_2_ (II:9;10) is the most prevalent strain in causing bacillary dysentery in developing countries. Chemical modifications such as glucosylation, O-acetylation, and phosphoethanolamine modifications of lipopolysaccharide (LPS) O antigen (Oag) contribute to the emergence of various serotypes. Sf6 is a *Shigella*-specific bacteriophage that infects only a limited range of *S. flexneri* serotypes [X, Y]. LPS Oag is the primary receptor for bacteriophage Sf6 where it uses its tailspike protein (TSP) in binding and hydrolysing LPS Oags. Sf6TSP has recently been shown to be capable of hydrolysing the LPS Oag of Type II strains, albeit modestly. Phage therapy has regained attention in recent years as an alternative therapeutic approach. Therefore, this study aimed to expand the host range of Sf6 to the prevalent *S. flexneri* serotype 2a_2_ strain. We discovered a new lytic Sf6 host range mutant that is capable of infecting *S. flexneri* serotype 2a_2_ and identified residues in Sf6TSP that may potentially be involved in binding and hydrolysing serotype 2a_2_ LPS Oag. This work increased the limited *Shigella*-specific bacteriophage collection and may be useful in the future for phage therapy and/or biocontrolling of *S. flexneri* in contaminated food and water.

## Teaser

Isolation of bacteriophage Sf6c mutant that infects *Shigella flexneri* serotype 2a_2_ strain, the most prevalent serotype that causes shigellosis.

## Introduction

*Shigella flexneri* is one of the most dominant strains in causing shigellosis in developing countries (Livio *et al*. 2014). Serotype 2a (29%), 3a (14%), and 1a (9%) are the three most prevalent serotypes in Asian countries (von Seidlein *et al*. 2006, Ye *et al*. 2010). Shigellosis is a global burden that has an annual morbidity rate of ∼269 million cases and ∼210 000 of mortality cases (Khalil *et al*. 2018). World Health Organisation has listed *Shigella* as a priority organism as it possesses great threat to human health (Tacconelli *et al*. 2018).

All *S. flexneri* serotypes except serotype 6 share the same O antigen (Oag) polysaccharide backbone that is composed of one N-acetylglucosamine (GlcNAc) residue and three L-rhamnose (Rha^I^-Rha^II^-Rha^III^) residues (Kenne *et al*. 1977). This forms the Oag polysaccharide of serotype Y. Various chemical modifications such as glucosylation, O-acetylation and phosphoethanolamine modification on different sugars increase Oag diversity and serotypes. *Shigella flexneri* serotypes are defined by a series of type and group O-factors (Boyd 1938, Carlin *et al*. 1989). Type O-factors are defined by Roman numerals (I, II, III, IV, V, VI, and VII) while group O-factors are defined by Arabic numerals (3,4; 6; 7,8; 9, and 10). Type O-factors are found within one serotype (e.g. serotype 2a and 2b share the type O-factor II) while group O-factors are shared among different serotypes (e.g. group factor 7,8 is found in serotype 2b, 3a, 5b, and X) (Carlin *et al*. 1989). The group O-factor 3,4 is defined as a backbone epitope and is shared by serotype 2a, 3b, 4a, 5a, and Y (Carlin and Lindberg 1987, Perepelov *et al*. 2012). The serotype Y strain has the antigenic formula of (-:3,4) because it does not have type O-factor.

*S. flexneri* 2457T (2a_2_) has two known Oag modifications, glucosylation on Rha^I^ (confers type O-factor II), and O-acetylations on Rha^III^ at position 3 or 4 (confers group O-factor 9) and on GlcNAc at position 6 (confers group O-factor 10), which are mediated by the *gtrIIBA* operon, *oacB*, and *oacD* genes, respectively (Kubler-Kielb *et al*. 2007, Perepelov *et al*. 2009, Sun *et al*. 2014, Wang *et al*. 2014). Therefore, the antigenic formula of *S. flexneri* 2457T is designated as (II:9;10). Both *gtrIIBA* operon and *oacD* gene are carried by the serotype-converting bacteriophage SfII prophage and are inserted between *proA* and *adrA* (Knirel *et al*. 2015). The *oacB* gene is carried by a transposon-like mobile element which is flanked by integrase and insertion sequences and is located upstream of *adrA* (Wang *et al*. 2014). Gene deletion of *oacD* only, *oacB* only or both *oacD oacB* in *S. flexneri* 2457T (2a_2_) gave rise to serotype 2a_3_ (II:9), 2a_1_ (II:10), and 2a (II:3,4), respectively, where serotype 2a_3_ and 2a_2_ are resistant to bacteriophage Sf6 (Teh *et al*. 2020).

Bacteriophage Sf6 is a short-tailed dsDNA virus that belongs to the family of *Podoviridae*. Sf6 is a temperate bacteriophage and is part of the subgroup ‘P22-like’ phages because the morphology of Sf6 is very similar to P22 (Casjens and Thuman-Commike 2011). Sf6 is known to infect serotype X and Y of *S. flexneri* strains, converting them into serotype 3a and 3b, respectively (Clark *et al*. 1991). Our recent study showed that Sf6 is also capable of infecting *S. flexneri* serotype 2a (II:3,4) and 2a_1_ (II:10) strains lacking the *oacB* gene, converting them into serotype 3b (III:6) and 3b_1_ (III:6;10), respectively (Teh *et al*. 2020). Sf6 recognises specific LPS Oag via its tailspike protein (Sf6TSP). Sf6TSP is an endorhamnosidase enzyme that binds reversibly to LPS Oag (primary receptor) (Kang *et al*. 2016) which facilitates the cleavage of Oag repeat units between the 1,3-α-linkage of two rhamnose residues, releasing an octasaccharide product (Lindberg *et al*. 1978, Chua *et al*. 1999). Hydrolysis of Oag repeat units brings Sf6 closer to the bacterial surface. Sf6 then binds irreversibly to OmpA or OmpC (secondary receptor) on the outer membrane (Parent *et al*. 2014).

Sf6TSP exists as a trimer (Muller *et al*. 2008) and consists of two domains, the highly conserved N-terminal domain (114 residues) that forms the capsid binding domain and the variable C-terminal domain (509 residues) that is responsible in recognising and cleaving the receptor into fragments (Steinbacher *et al*. 1997, Chua *et al*. 1999, Casjens *et al*. 2004). The enzymatic active sites (residues E366 and D399) of Sf6TSP are located on two different subunits of a trimer (Muller *et al*. 2008). Hence, a trimeric Sf6TSP contains three independent elongated glycan binding sites for LPS Oag fragments (Kunstmann *et al*. 2020). Sf6TSP has been shown to interact with LPS Oag of serotype 2a weakly (Kunstmann *et al*. 2018) and we have recently shown that Sf6TSP is capable of modestly cleaving LPS Oag repeat units of serotype 2a_1_, 2a_2_, 2a_3_, and 2a strains (Teh *et al*. 2020).

To date, there are no *Shigella* vaccines generally available, and the emergence of multi-antibiotics resistance strains have necessitated a need to seek alternative methods of treatment. Phage therapy has regained attention in recent years to treat diseases caused by multi-antibiotic resistance bacteria. This study aims to isolate a Sf6 host range mutant that is capable of infecting the most prevalent *S. flexneri* strain.

A new Sf6 host range mutant that infects *S. flexneri* serotype 2a_2_ was isolated in this study. Amino acid residues that are potentially involved in binding and hydrolysing LPS Oag of serotype 2a strains were also identified in this study. Our study contributes to understanding of the interaction between bacteriophage Sf6 and *S. flexneri* serotype 2a.

## Materials and methods

### Bacterial strains and bacteriophage

The strains used in this study are listed in Table 1. Sf6c is a clear plaque derivative of Sf6 and was propagated on *S. flexneri* serotype Y PE577 (Morona *et al*. 1994).

**Table 1.** Strain used in this study.

Strain	Relevant characteristics	Reference
2457T	Virulence plasmid positive *S. flexneri* serotype 2a_2_	Lab collection
PE577	*S. flexneri* serotype Y	Lab collection
RMA2159	Virulence plasmid-cured *S. flexneri* serotype 2a_2_	Lab collection
RMA4266	*S. flexneri* serotype 5b (PE565)	Lab collection
RMA4267	*S. flexneri* serotype 4a (PE566)	Lab collection
RMA4269	*S. flexneri* serotype 3b (PE645)	Lab collection
RMA4270	*S. flexneri* serotype 2b (PE824)	Lab collection
RMA4271	*S. flexneri* serotype 3a (PE843)	Lab collection
RMA4272	*S. flexneri* serotype 4b (PE572)	Lab collection
RMA4274	*S. flexneri* serotype 1a (PE839)	Lab collection
RMA4275	*S. flexneri* serotype 1b (PE840)	Lab collection
RMA4278	*S. flexneri* serotype 5a (PE856)	Lab collection
RMA4335	*S. flexneri* serotype X (PE576)	Lab collection
MYRM1069	*S. flexneri* 2457T Δ*oacB* serotype 2a_1_	Teh *et al*. 2020
MYRM1071	*S. flexneri* 2457T Δ*oacD* Δ*gtrIIBA* serotype Y_1_	Teh *et al*. 2020
MYRM1091	*S. flexneri* 2457T Δ*oacD* serotype 2a_3_	Teh *et al*. 2020
MYRM1124	*S. flexneri* 2457T Δ*oacD* Δ*oacB* serotype 2a	Teh *et al*. 2020
MYRM1134	*S. flexneri* 2457T Δ*gtrIIBA* Δ*oacB* serotype Y_3_	Teh *et al*. 2020
MYRM1136	*S. flexneri* 2457T Δ*gtrIIBA* serotype Y_2_	Teh *et al*. 2020
MYRM1138	*S. flexneri* 2457T ΔAll_ΔDIIBA+B_ serotype Y	Teh *et al*. 2020

### Growth media and growth conditions

All strains used in this study were routinely grown in Lysogeny Broth (LB) medium. Bacterial cultures were cultured for 18 h, diluted 1:20 and grown for 4 h with aeration at 37°C.

### Bacteriophage plaque assay and spot assay

The bacteriophage plaque assay was performed by mixing 100 µL of bacteriophage (10^–8^ of an approximately 1 × 10^11^ pfu mL^–1^ stock) with equal amount of bacterial culture and incubated for 4 min at RT followed by 1 min incubation at 45°C. Then, 3 mL of soft agar (0.75% [w/v] LB agar, prewarmed at 45°C) was added into the bacterial/phage mixture and immediately overlayed onto a 25 mL LB agar plate. The plate was incubated at 37°C for 18 h.

For the bacteriophage spot assay, 15 µL of bacterial culture was mixed with 0.35 mL of soft agar (0.75% [w/v] LB agar, prewarmed at 45°C) and immediately overlayed onto a 3 mL LB agar in a 12-well tray. Once the soft agar overlay was dried, 5 µL of phage (undiluted phage stock) was spotted onto the overlay and let dry. The plate was incubated at 37°C for 18 h.

### DNA manipulation

The *gp14* gene (encodes TSP) of Sf6(2a)c was PCR amplified and subsequently sequenced with primers MY_228_Up_tsp_F (5′ cttcgcaaacaccaggaac 3′) and MY_229_Down_tsp_R (5′ gttaagtgccgtgactgtt 3′) that target upstream and downstream of *gp14*, respectively. DNA sequence was analysed by Australian Genome Research Facility (Adelaide, Australia).

### Expansion of Sf6c host range mutant

To isolate a new host range mutant that form clear plaques on *S. flexneri* serotype 2a_2_, Sf6c was first propagated in co-culture of PE577 and *S. flexneri* serotype 2a_2_ RMA2159 (ratio 1:1) for multiple rounds. Briefly, 100 µl of Sf6c (7.8 × 10^10^ pfu mL^–1^) was added into 1:20 of co-culture and grown at 37°C for 24 h with aeration. The co-culture was then pelleted, supernatant that contained phage was transferred to a new glass bottle and treated with chloroform for 30 min at 37°C to remove any live bacteria. One hundred microliters of the supernatant was then mixed with co-culture and the propagation repeated. After 8 rounds of propagation, a plaque assay was performed with RMA2159. Phage from a single clear plaque that formed on RMA2159 was purified, and further propagated on RMA2159 as described above. The newly isolated Sf6c host range mutant was named Sf6(2a)c.

### Preparation of LPS samples and LPS analysis

The equivalent of 1 × 10^9^ bacteria were centrifuged (2200 × *g*, 1 min) and resuspended in 50 µL lysis buffer, heated at 100°C for 10 min, cooled and treated with 2.5 µg mL^–1^ Proteinase K (#AM2542, Thermo Fisher) at 56°C for at least 4 h. The LPS samples were then subjected to SDS-PAGE and silver staining (Teh *et al*. 2012).

### Preparation of formalin-fixed bacteria

The equivalent of 1 × 10^9^ bacteria were centrifuged (2200 × *g*, 1 min), washed once with PBS and fixed with 100 µL of 1% (v/v) formalin in PBS at RT for 45 min. The formalin-fixed bacteria were washed once with PBS and resuspended in 100 µL PBS.

### LPS hydrolysis assay by bacteriophage

The formalin-fixed bacteria (1 × 10^9^ bacteria mL^–1^) were centrifuged (2200 × *g*, 1 min), resuspended in 100 µL of bacteriophage (1 × 10^11^ pfu mL^–1^) and incubated at 37°C for 45 min. The treated bacteria were washed twice with Milli-Q water and prepared LPS samples as described above.

## Result and discussion

### Sf6 expanded its host range to serotype 2a_2_ and 2a_3_

Sf6c is a derivative of Sf6 that forms clear plaques (lytic) on *S. flexneri* serotype Y (Morona *et al*. 1994). We and others have recently shown that Sf6TSP has some weak interaction with LPS Oag of serotype 2a strains (Kunstmann *et al*. 2018) and Sf6TSP is capable of hydrolysing LPS Oag of all serotype 2a strains (Teh *et al*. 2020). Therefore, we hypothesised that propagation of Sf6c in a *S. flexneri* co-culture that contained PE577 (serotype Y; susceptible to Sf6c) and RMA2159 (serotype 2a_2_; resistant to Sf6c) would allow Sf6c to evolve and improve its interaction with serotype 2a strains. RMA2159 was used as it is a virulence plasmid-cured *S. flexneri* 2457T strain and was safer to handle during phage propagation. The process of isolating Sf6c host range mutant was described in the Methods. Sf6c that couldn't plaque on RMA2159 (2a_2_) initially started forming opaque plaques after few rounds of propagation in co-cultures (data not shown) and eventually formed clear plaques on RMA2159 after eight rounds of propagation in co-cultures. We named the newly isolated host range mutant of Sf6c as ‘Sf6(2a)c’.

**Figure 1.**
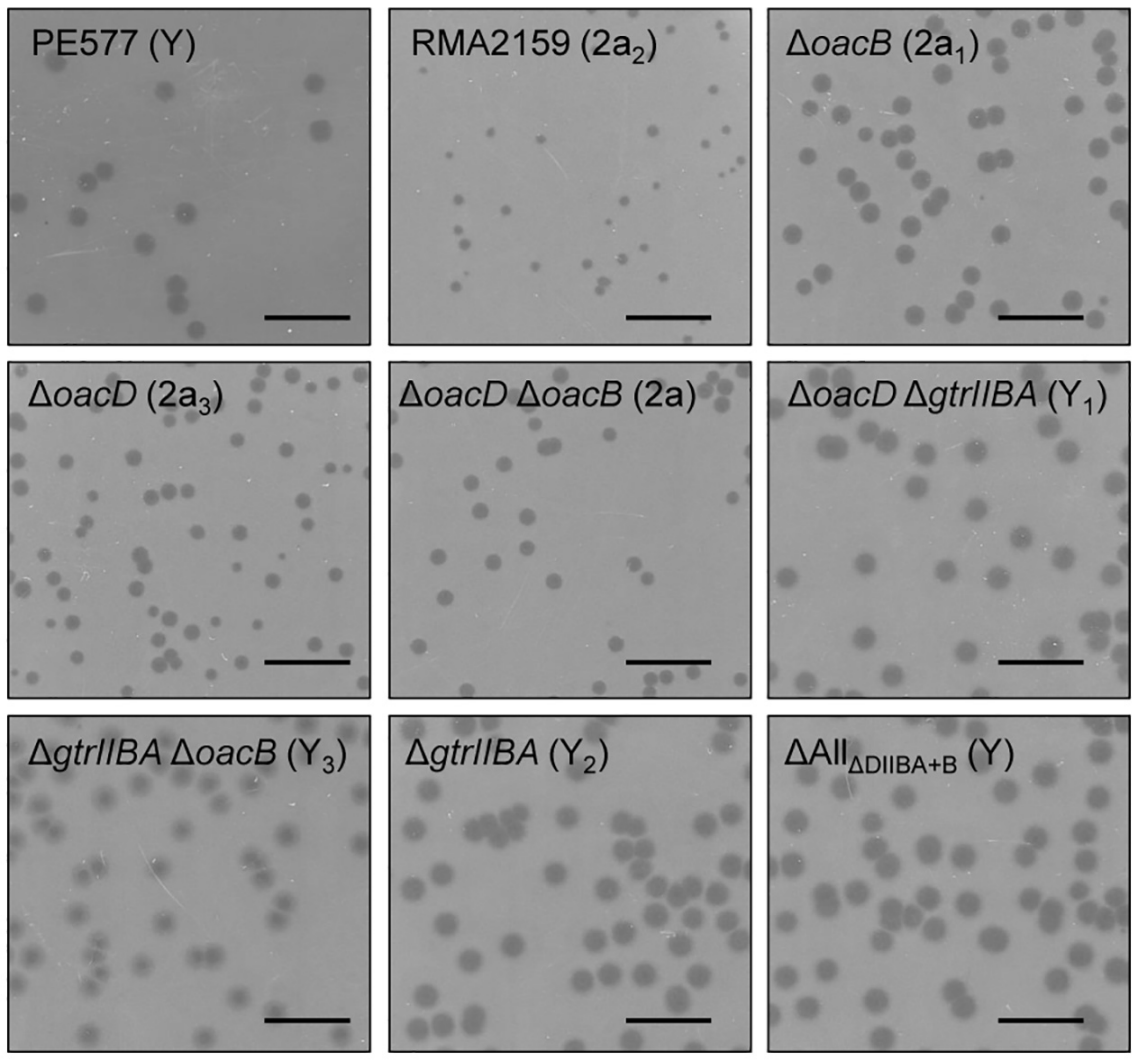
Sensitivity of serotype Y and 2a *S. flexneri* strains to the new host range mutant Sf6(2a)c. Bacteriophage Sf6(2a)c plaque assay. About 100 µL of overnight cultures of *S. flexneri* were incubated with 100 µL Sf6(2a)c (10^–8^) prior to mixed with 3 mL of soft LB agar and overlayed onto 25 mL LB agar plates. The plates were incubated at 37°C, 18 h. Serotypes of each strain are indicated in parentheses. Scale bar indicates 10 mm.

Sf6(2a)c plaque assay results showed that Sf6(2a)c was capable of infecting PE577 (Y), RMA2159 (2a_2_), and all isogenic serotype variant strains of 2457T, such as ΔAll_ΔDIIBA+B_ (Y), Δ*oacD* Δ*gtrIIBA* (Y_1_), Δ*gtrIIBA* (Y_2_), Δ*gtrIIBA* Δ*oacB* (Y_3_), Δ*oacB* (2a_1_), Δ*oacD* Δ*oacB* (2a) and Δ*oacD* (2a_3_) strains (Fig. 1). Serotype 2a_2_ and 2a_3_ strains that had previously been shown to be resistant to Sf6c infection were now susceptible to Sf6(2a)c (Teh *et al*. 2020). This indicated that Sf6(2a)c had evolved and capable of infecting *S. flexneri* serotype 2a strains that were resistant to Sf6c.

### Sf6(2a)c retained its ability to infect serotype Y and X

**Figure 2.**
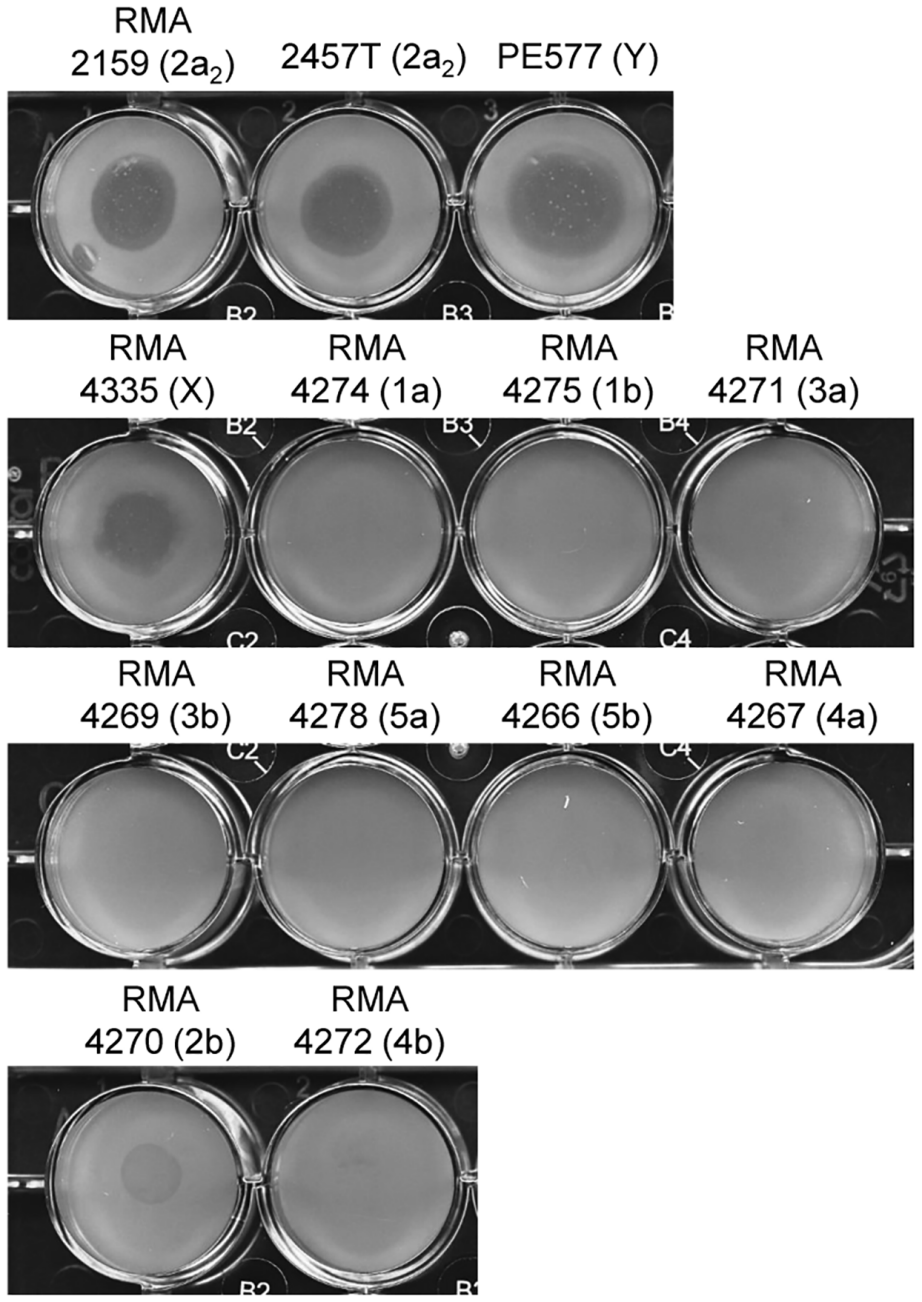
Sf6(2a)c spot assay on *S. flexneri* strains with different serotypes. Fifteen microliters of overnight cultures of *S. flexneri* were mixed with 350 µL of soft LB agar, overlayed onto 3 mL LB agar in a 12-well tray and let dry. About 5 µL of undiluted Sf6(2a)c phage stock was then spotted onto the soft agar. The tray was incubated at 37°C for 18 h. Serotypes of each strain are indicated in parentheses.

We found that Sf6(2a)c was still capable of infecting serotype Y strains. We then investigated if Sf6(2a)c was still able to infect serotype X (like Sf6c) and/or had expanded its host range to other serotypes. A spot assay was performed on various serotypes strains (Y, X, 1a, 1b, 2a_2_, 2b, 3a, 3b, 5a, 5b, 4a, and 4b) (Fig. 2). Sf6(2a)c only formed clear spots on the serotype X strain, and hence Sf6(2a)c retained its ability to infect serotype X. Surprisingly, Sf6(2a)c formed an opaque spot on serotype 2b strain (RMA4270), indicating that the growth of serotype 2b strain was affected (Fig. 2). However, Sf6(2a)c could not form plaques on the serotype 2b strain (data not shown), suggesting that Sf6(2a)c has extremely weak interaction with serotype 2b strain and is only able to affect the growth of serotype 2b at high titre. The serotype 2b LPS Oag has glucosylation on both Rha^I^ and Rha^III^ residues, while serotype 2a_2_ and 2a_3_ LPS Oags have both glucosylation and O-acetylation on Rha^I^ and Rha^III^, respectively (Teh *et al*. 2020). A recent study reported that O-acetylation or glucosylation on Rha^III^ had a similar effect on Oag helical conformation (Hlozek *et al*. 2020). Therefore, Sf6(2a)c which has the ability to infect 2a_2_ and 2a_3_ strains may then be able to interact with the LPS Oag of serotype 2b, albeit weakly.

### Sf6(2a)c tailspike protein has mutations that correlate to infection of serotype 2a_2_ and 2a_3_ strains

**Figure 3.**
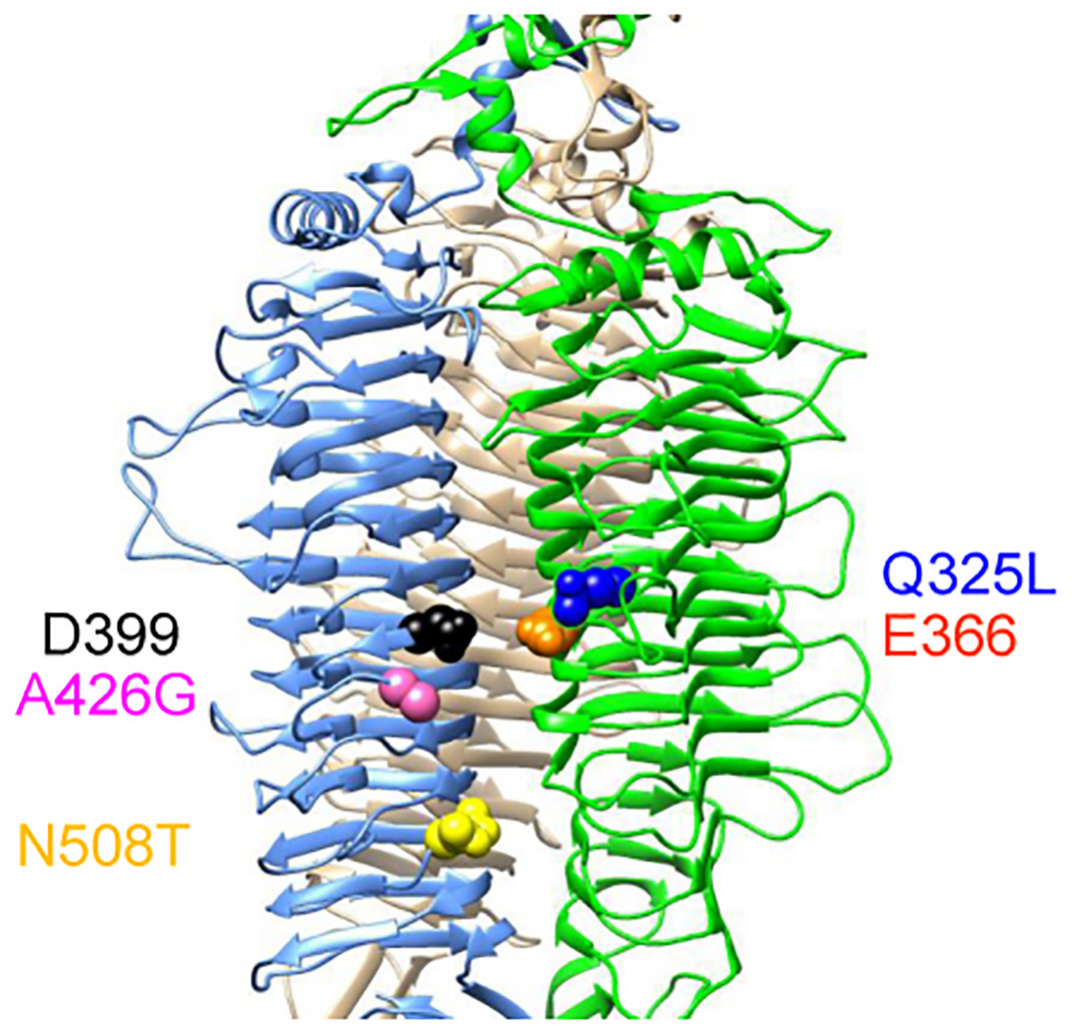
Location of mutated residues in Sf6(2a)cTSP. Three subunits of native TSP (PBD: 2VBK) are illustrated in light blue, green and grey cartoon. The catalytic residues, D399 and E366 are shown in black and orange spheres, respectively. The mutated residues identified in Sf6(2a)cTSP are depicted in blue (Q325L), pink (A426G), and yellow (N508T) spheres.

TSP (encoded by *gp14*) is the protein that mediates binding of Sf6 to LPS Oag of *S. flexneri*. Therefore, we hypothesised that Sf6(2a)c has mutated residues within the binding groove of TSP in order to increase its binding affinity to LPS Oag of serotype 2a_2_ and 2a_3_ strains. Sequencing of the Sf6(2a)c *gp14* gene revealed three amino acids change: Q325L, A426G and N508T. Coincidentally, these amino acids mutations are in close proximity to the enzyme's active site residues (E366 D399), where both Q325 L and A426G are located within the glycan binding groove while N508T is located below the groove (Fig. 3). In a trimeric TSP, Q325 L is located on the same subunit as E366, while A426G and N508T are located on another subunit like D399 (Fig. 3). A recent MD simulations study reported that two Sf6TSP E366A D399A variants (V204C and S246C) that are in close proximity to the Oag binding site, had a slight increase in binding affinity to serotype Y LPS Oag (Kunstmann *et al*. 2020). This indicated that Sf6TSP can be engineered to increase its binding affinity to LPS Oag. In Sf6(2a)c, its TSP has mutated as a result of adaptation to improve its interaction with LPS Oag of serotype 2a_2_ and 2a_3_. The adaptive mutational alterations in the *tsp* gene arose spontaneously, and the sequences are not present in the genomes of the host strains.

### Sf6(2a)cTSP has improved serotype 2a LPS Oag hydrolysis

**Figure 4.**
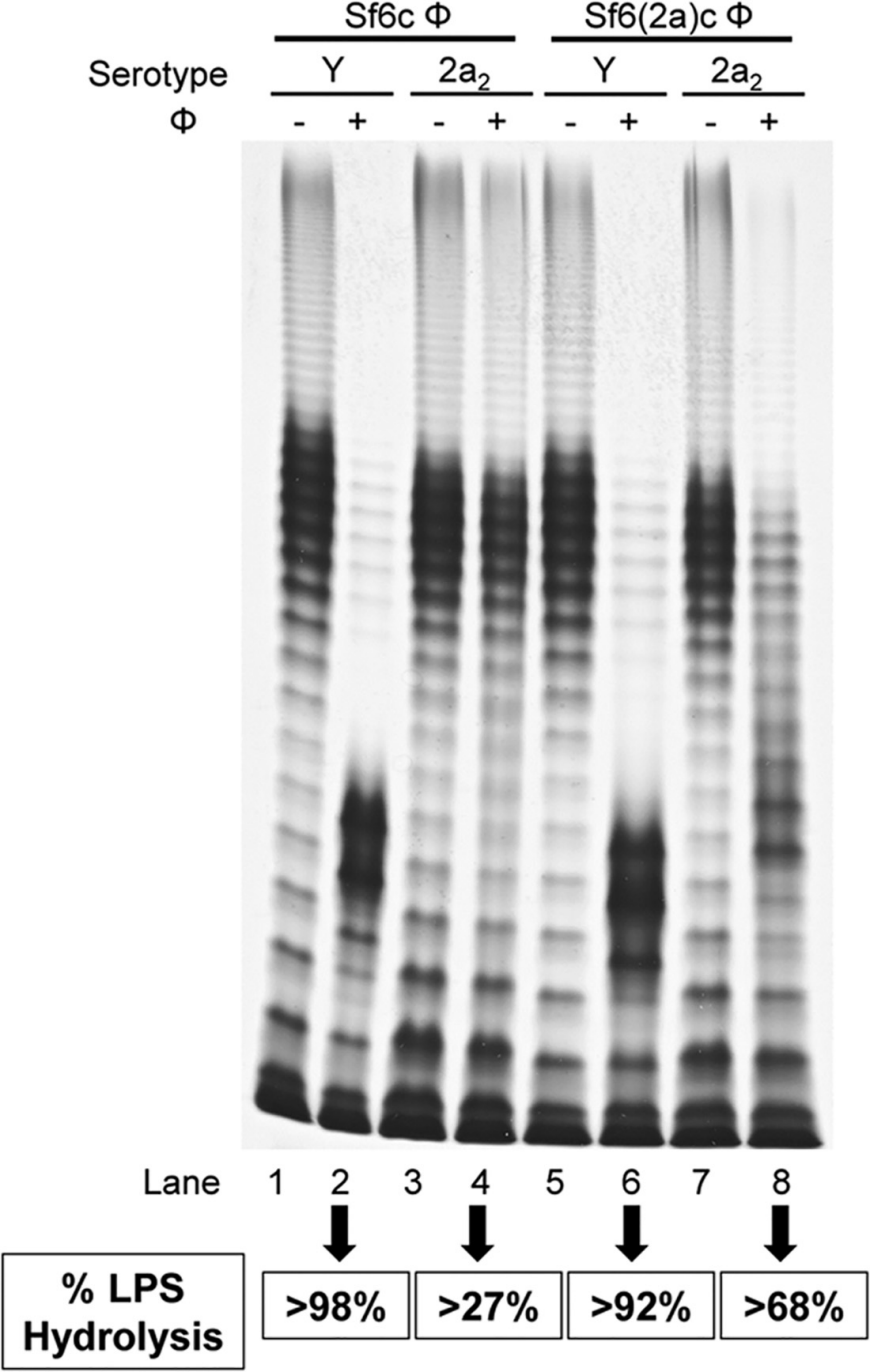
LPS hydrolysis assay mediated Sf6c or Sf6(2a)c bacteriophage on *S. flexneri* serotype Y (PE577) and 2a_2_ (RMA2159) strains. Overnight cultures of *S. flexneri* (1 × 10^9^ bacteria mL^–1^) were formalin-fixed for 45 min at RT, washed twice with PBS and resuspended in 100 µL of bacteriophage (1 × 10^11^ pfu mL^–1^). The mixture was incubated at 37°C for 45 min, washed twice with Milli-Q water and resuspended in 50 µL lysis buffer. LPS samples were then subjected to SDS-PAGE and LPS silver staining as described in the Methods. The untreated and bacteriophage-treated LPS Oag repeat units 11–17 were measured by densitometry using ImageLab 6.1 and the results are presented as percentage of LPS hydrolysis/reduction.

We have previously shown that while Sf6TSP can hydrolyse serotype 2a_2_ LPS Oag, it does so modestly, in a dose-dependent manner (Teh *et al*. 2020). We then investigated if the Sf6(2a)cTSP was able to hydrolyse serotype Y (PE577) and 2a_2_ (RMA2159) LPS Oags. We initially attempted to overexpress and purify Sf6(2a)cTSP for use in LPS hydrolysis assays but were unsuccessful due to low yield and stability. Therefore, LPS hydrolysis assays were performed on formalin-fixed bacteria using Sf6c and Sf6(2a)c phage. The results were analysed with SDS-PAGE and silver staining (Fig. 4). Oag repeat units 11–17 of the untreated and Sf6c- or Sf6(2a)c-treated LPS Oags of each strain were quantified by densitometry and the results were presented as a percentage of LPS Oag reduction or hydrolysis in Fig. 4.

As expected, Sf6c-mediated LPS hydrolysis of PE577 (Y) and RMA2159 (2a_2_) were > 98% and > 27%, respectively (Fig. 4, Lane 2 and 4, respectively). This is consistent with our previously reported data by using Sf6TSP protein (Teh *et al*. 2020). This is also similar for Sf6(2a)c-mediated LPS hydrolysis where serotype Y LPS Oag had > 92% hydrolysis (Fig. 4, Lane 6). Excitingly, there was a significant increase in Sf6(2a)c-mediated LPS hydrolysis of RMA2159 (> 68%), in comparison to Sf6c-mediated LPS hydrolysis (> 27%) of the same strain (Fig. 4, Lane 8 and 4, respectively). This data suggested that the amino acids mutations within the Sf6(2a)cTSP protein enhanced LPS hydrolysis of serotype 2a LPS Oags and subsequently allowing infection of 2a strains by Sf6(2a)c phage. Notably, Sf6(2a)c-mediated LPS hydrolysis on RMA2159 was incomplete and this is consistent with our previous finding that Sf6TSP was not able to achieve complete hydrolysis on LPS Oag of *S. flexneri* 2457T-derived strains (Teh *et al*. 2020). The data also suggested that partial hydrolysis of LPS Oags is sufficient for Sf6(2a)c to infect a serotype 2a strain.

## Conclusion

A new host range mutant of Sf6c that infects the most prevalent *S. flexneri* serotype (2a_2_) was successfully isolated in this study. We showed that Sf6(2a)cTSP had improved LPS hydrolysis ability on serotype 2a_2_ strain and infection. The isolation of Sf6(2a)c contributes to our understanding of the interaction between Sf6TSP and its Oag receptor. Sf6(2a)c may be useful for phage therapy or biocontrolling of *S. flexneri* in contaminated food or water.
